# Kikuchi-Fujimoto Disease: A Rare Benign Cause of Lymphadenopathy That Mimics Malignant Lymphoma

**DOI:** 10.7759/cureus.23177

**Published:** 2022-03-15

**Authors:** Israa Gism Elseed, Haitham Osman, Osman Ahmedfiqi, Fatima Najmi, Abdulqader Al-Hebshi

**Affiliations:** 1 Medicine, Prince Mohammed bin Abdulaziz Hospital, Ministry of National Guard Health Affairs, Madinah, SAU; 2 Hematology, Prince Mohammed bin Abdulaziz Hospital, Ministry of National Guard Health Affairs, Madinah, SAU; 3 Pathology, Prince Mohammed bin Abdulaziz Hospital, Ministry of National Guard Health Affairs, Madinah, SAU; 4 Pediatric Hematology-Oncology, Prince Mohammed bin Abdulaziz Hospital, Ministry of National Guard Health Affairs, Madinah, SAU; 5 Pediatrics, King Saud Bin Abdulaziz University for Health Sciences, Riyadh, SAU

**Keywords:** lymphoma, lymph node., histopathology, histiocytic necrotizing lymphadenitis, kikuchi-fujimoto disease

## Abstract

Kikuchi-Fujimoto disease (KFD), also called histiocytic necrotizing lymphadenitis, is a benign self-limited disease involving lymph node enlargement with unclear causes and high fever. It was first seen in Japan and has wide array of differentials, and thus it can be confused with other causes of lymphadenitis leading to an incorrect treatment. We describe a case of a 34-year-old man with prolonged painless cervical lymphadenopathy and fever, in whom KFD, that is histiocytic necrotizing lymphadenitis, was diagnosed after the gold standard test of lymph node biopsy. Physical examination, history, and other relevant investigations were performed to rule out infectious and autoimmune causes. For management, prednisone 80mg and hydroxychloroquine were given, which lead to an uneventful full recovery. Our findings were compared to other similar studies conducted in the past. KFD is a self-limited condition that usually resolves by itself. KFD should be considered in the differential diagnosis of lymphadenopathy. An accurate diagnosis requires close collaboration between clinicians and pathologists. Early diagnosis of KFD allows reducing stress caused by alarming symptoms and avoiding unnecessary treatment.

## Introduction

Fever with lymphadenopathy has multiple differentials and possible diagnoses. Kikuchi-Fujimoto disease (KFD) or histiocytic necrotizing lymphadenitis is a very rare cause of lymphadenopathy, most often cervical [1, 2]. It is a self-limited condition, especially characterized by benign lymphadenopathy along with systemic disorder involving lymph nodes (lymphadenopathy) with unknown origin [2, 3, 4]. KFD was first described in Japan in 1972 by two independent researchers, Kikuchi and Fujimoto [3,5-7]. Since then, it has been reported and named globally. It usually affects a young population (younger than 40 years), but it can also be reported in any age group [3,5]. Some of the research studies from Asian countries suggest that the male-to-female ratio is closer to 1:1 [6,7]. KFD is characterized by localized lymphadenopathy, with cervical lymph nodes being the most commonly affected [8,9]. The onset is typically subacute or acute with a short course of symptoms [6-9].

Nevertheless, KFD has been already described as a reason behind pyrexia of unknown origin (PUO). Other symptoms are less frequent, including chills, night sweats, arthralgia, and weight loss, along with diarrhea and nausea [8,10]. Involvement of the posterior cervical group is the most common feature [10]. However, all areas can be involved. Usually, lymph nodes appear painful and tender and are of moderate size.

Atypical presentations and extramural involvements are possible, mainly cutaneous manifestations and aseptic meningitis [10]. Generalized forms are sometimes associated with a splenomegaly or hepatomegalies that have been described previously [10]. Involved lymph nodes demonstrate para-cortical areas of apoptotic necrosis with abundant karyorrhectic debris and proliferation of histiocytes plasmacytoid dendritic cells and CD8 (+) T cells in the absence of neutrophils and eosinophils.

KFD is confirmed as a diagnosis when clinical and histologic characteristics overlap as well as the multiple therapeutic approaches. Treatment consists of supportive steps, and long-term follow-up is recommended if an association with systemic lupus erythematosus (SLE) is reported, including arthralgia and weight.

## Case presentation

A 34-year-old Saudi male with hypertension only presented to our hospital with enlarged right-sided cervical swelling (lymphadenopathy), which was painless and had appeared four weeks before his presentation. The swelling was associated with a high-grade intermittent fever, malaise, vomiting, anorexia, and weight loss of 6 kg over this period. He denied any symptoms of upper or lower respiratory tract infections or gastrointestinal or urinary complaints. He was advised a five-day course of oral antibiotic amoxicillin but had no improvement.

The patient also reported that a similar attack occurred 12 years back when he had a febrile illness with cervical swelling that was investigated thoroughly; no diagnosis was concluded at that time. He was treated empirically with antipyretic and anti-inflammatory medication. It resolved within eight weeks, but no detailed medical reports were available. His family history revealed no similar condition, no contact with tuberculosis (TB) patients, nor any other infectious diseases. He denied any history of recent international travel, and he reported that there was no history of extramarital sexual relationships.

**Figure 1:**
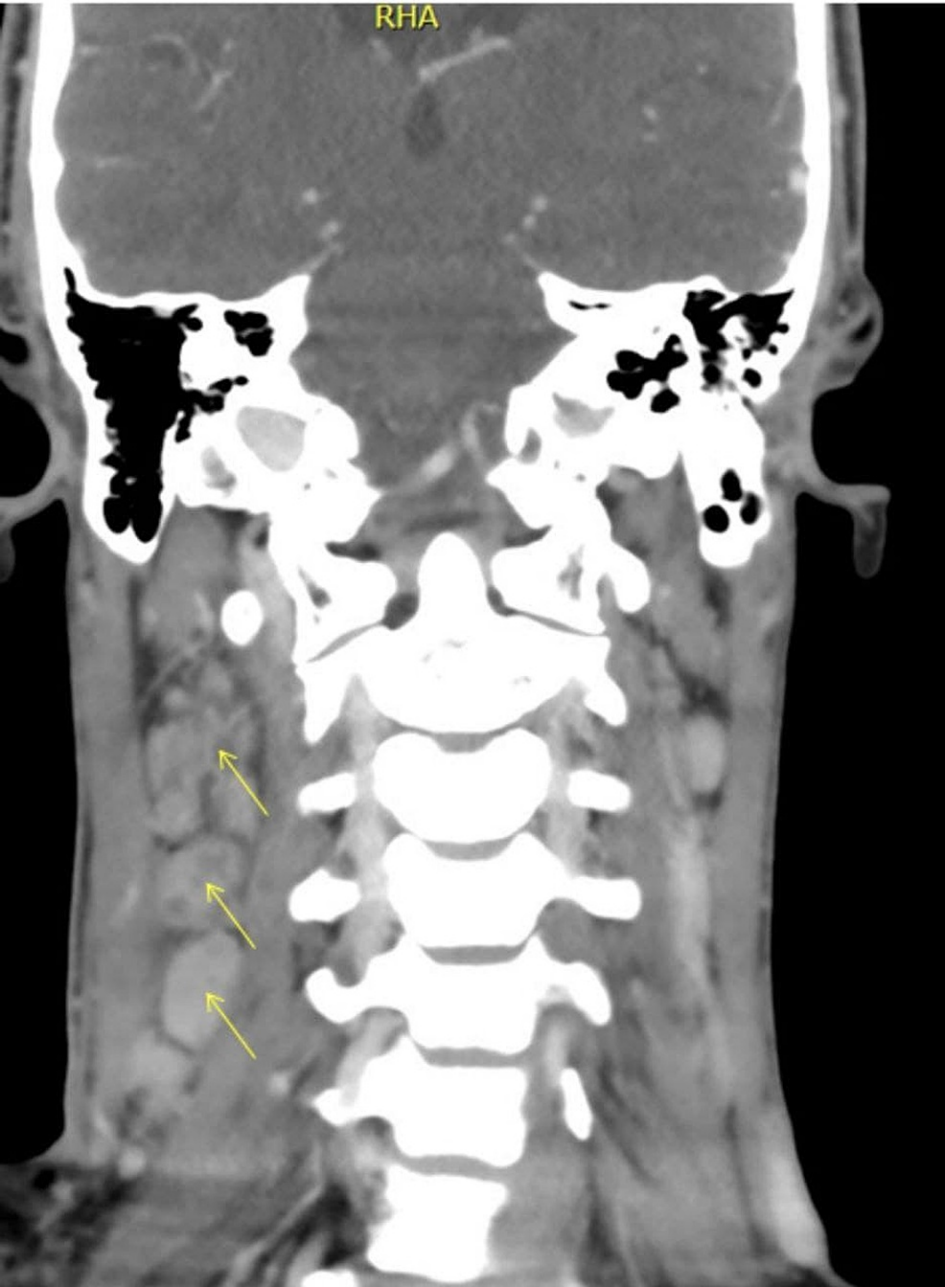
Neck CT: sagittal section of right deep cervical lymphadenopathy with central necrosis.

**Figure 2:**
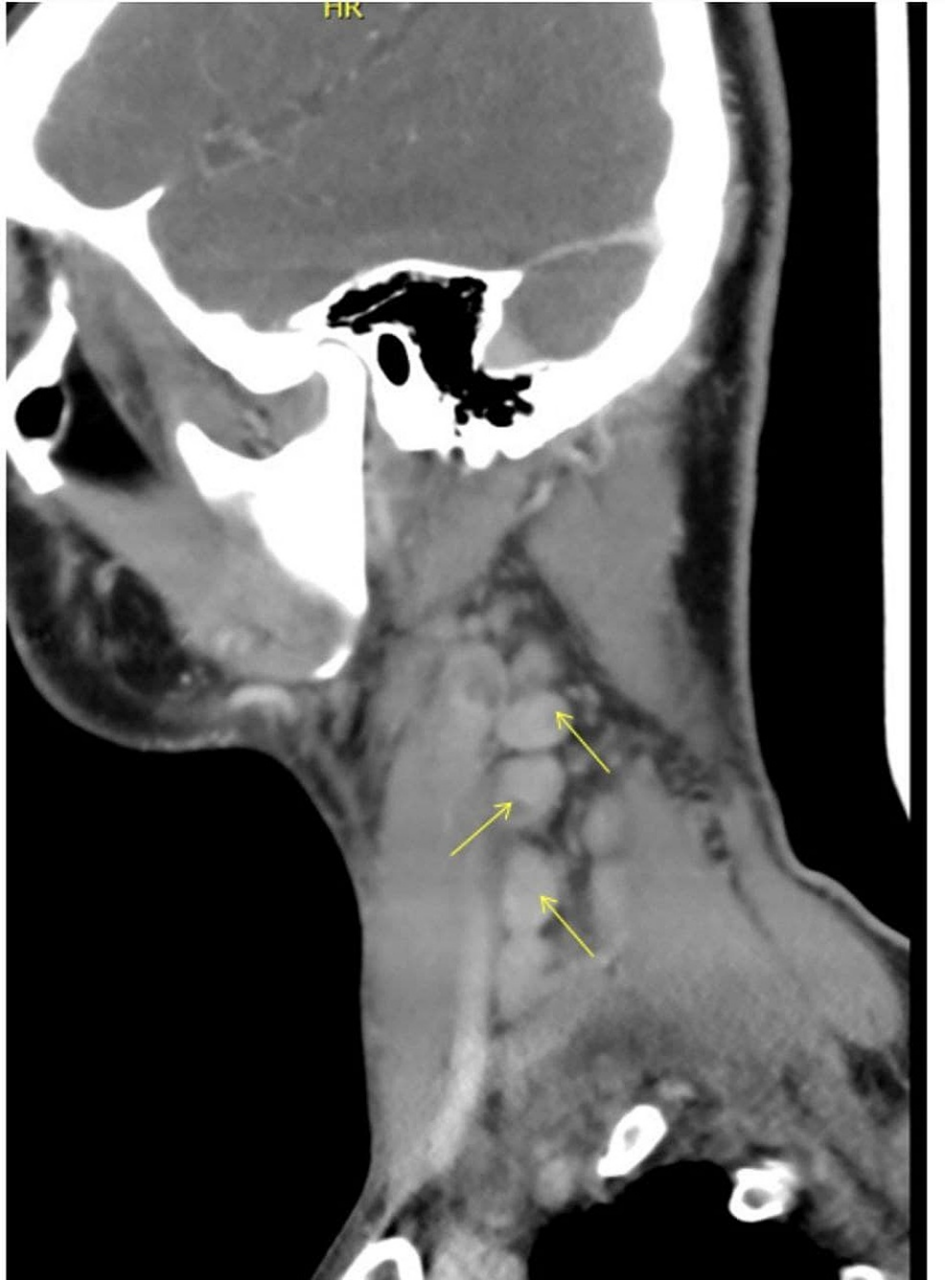
Neck CT: lateral sagittal view of right deep cervical lymphadenopathy.

His physical examination on admission revealed an average body built male without jaundice. He was febrile with a temperature of 38.1°C. His throat was not congested, and there was no ear discharge. There was a right cervical palpable enlarged lymph node. The largest node was in the posterior triangle, measuring 3cmx4cm; it was firm, non-tender, mobile, not attached to deep structure, with no discharging sinuses over it. The right supraclavicular lymph node was also palpable. Axillary and inguinal lymph nodes were not detectable. His abdominal, cardiovascular, respiratory, skin, and CNS examination findings were normal. CT scan of his neck confirmed multiple right-sided deep cervical lymphadenopathies in the right posterior triangle and deep jugular chain, where the largest one measured about 33mm with central necrosis/caseation (Figures 1, 2). The whole-body CT scan with contrast did not report splenomegaly, hepatomegaly, or hidden abscess collection, and there was no other lymphadenopathy apart from the aforementioned cervical one.

The workup for lymphadenopathy was performed and included complete blood cells (CBC), which was normal, and serological tests such as antinuclear antibodies (ANA), rheumatoid factor, anti-double-stranded DNA (dsDNA), anti-Ss-A, anti-SS-B, anti-SM, and complement, Brucella, toxoplasma, leishmanial, cytomegalovirus (CMV), and Epstein-Barr virus (EBV) were also negative. The results of bacteriological cultures and acid-fast bacilli (AFB) smear were negative as well. Echocardiogram was normal.

**Figure 3:**
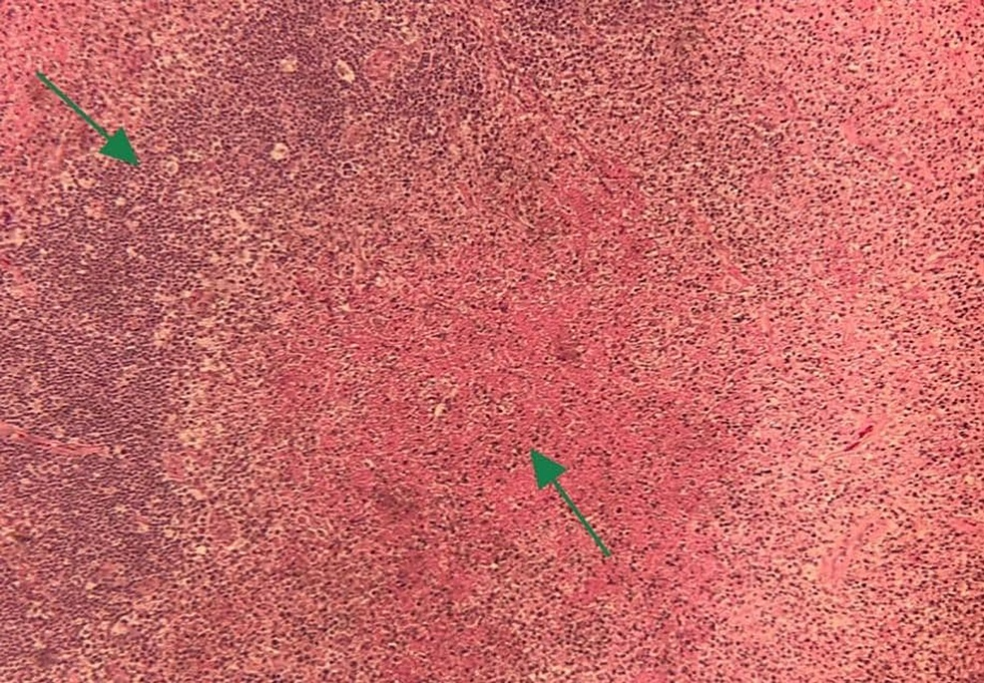
At high magnification, the area shows lymphoid tissue next to geographic necrosis.

**Figure 4:**
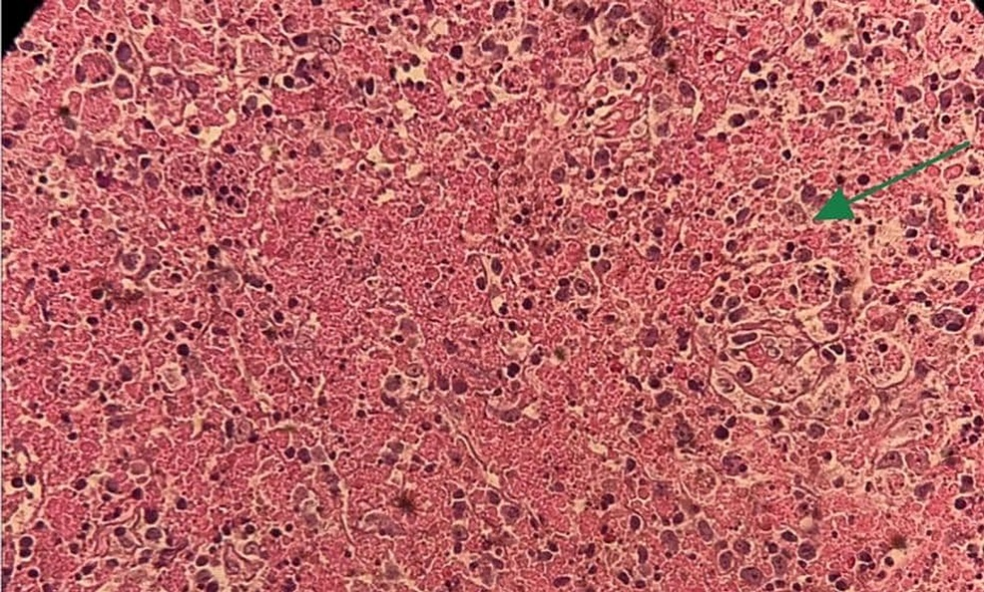
Histiocytes and lymphocytes but lack neutrophils or eosinophils.

He underwent cervical lymph node excisional biopsy, which was reported 10 days later and showed para-cortical irregular infiltration of histiocytes mixed with many apoptotic cells with abundant karyorrhectic nuclear debris, small mature lymphocytes, plasma cells, plasmacytoid cells (mostly plasmacytoid dendritic cells), and large areas of necrosis (Figure 3). A polymorphic population of lymphoid and histiocytes cells, including small and medium-sized lymphocytes and immunoblasts, rimmed these areas. No neutrophils or eosinophils were reported (Figure 4). The infiltrating histiocytes were positive for CD68, CD163, CD11c, and myeloperoxidase, and negative for S100 and CD1a. They also showed increased index by Ki-67 immune stain (up to 50%). The surrounding lymphoid cells were mainly T-cells. Most lymphoid cells expressed BCL2 and lacked expression of CD10 and BCL6.

Based on histopathological findings, KFD was diagnosed. The patient was started on oral hydroxychloroquine 200mg twice a day along with oral prednisone (1 mg/kg) that was tapered over six weeks. The fever subsided within a few days, and his lymphadenopathy disappeared over the first month. Hydroxychloroquine was stopped after four months, and the patient continued to feel well on his six-month follow-up post-treatment period. He was followed in the clinic for two years, and now he is in remission.

## Discussion

KFD is defined as histiocytic necrotizing lymphadenitis of unexplained origin [11]. It is an uncommon cause of lymphadenopathy. It is characterized by localized lymphadenopathy mainly affecting cervical lymph nodes, and a rare incidence in axillary and supraclavicular lymph nodes has been reported. Tenderness of the lymph node may or may not be present. Fever, fatigue, and joint pain are the other most frequent findings. As seen in our observation, laboratory studies were non-specific. A common finding is leukopenia and atypical lymphocytes; however, the majority of patients have a normal CBC count. Erythrocyte sedimentation rate (ESR) is increased in most patients, and there may be abnormalities in liver enzymes and raised lactate dehydrogenase (LDH) [11]. Our patient had fever, painless cervical lymphadenopathy, arthralgia, leukopenia, anemia, and a high ESR.

The natural course of this disease and physical symptoms seen in this patient were in line with that reported in the literature [1]. But the histopathological findings and B-type symptoms observed in our patient mimicked those seen in malignant conditions such as lymphoma. The preliminary report was consistent with the diagnosis of peripheral T-cell lymphoma not otherwise specified (PTCL-NOS-Type). However, there were some unusual features, such as diffusely effaced lymph node architecture by abnormal lymphoid proliferation exhibiting large areas of geographic necrosis and a starry sky appearance with sheets of foamy histiocytes. For that reason, a second opinion and further immune stains were applied to the tissue sample, such as CD123 performed to highlight the plasmacytoid dendritic cell population within the infiltrate, which confirms the diagnosis of KFD.

The differential diagnosis of KFD includes lymphoma, TB, SLE, blastic plasmacytoid dendritic cell neoplasm, Kawasaki's disease, sarcoidosis, herpes simplex virus (HSV), EBV, and metastatic adenocarcinoma [12]. The clinical picture of KFD may mimic several diagnoses such as infectious mononucleosis and TB, and even more serious diagnoses such as lymphomas, which may lead to diagnostic delay or misdiagnosis, especially in TB endemic areas such as the Saudi population. But the lack of our patient’s contact with any infectious disease helped us to rule out these possibilities. In addition to previous differential diagnoses, necrotizing lymphadenitis and SLE showed similar histologic features; however, a diagnosis of SLE was not supported clinically in this case as the patient did not meet the clinical criteria for a diagnosis of SLE. The absence of hematoxylin bodies and the Azzopardi effect also made it less likely. It was also excluded based on negative serological tests. The distinction of KFD from SLE is difficult, but the absence or paucity of hematoxylin bodies, plasma cells, and neutrophils suggest KFD.

Lymph node biopsy results provided a clearer picture. The characteristic histological findings of KFD are single or multiple areas within the lymph node that demonstrate necrosis and histiocytic cellular infiltrates. The capsule of the node may be invaded, and perinodal inflammation is common [13]. The histological features of the lymph node biopsy of our patient revealed the presence of numerous atypical monocytes and T-cell immune blasts and extensive areas of necrosis with a predominant histiocytic component. The conclusive final report was that the overall pathological findings are consistent with necrotizing histiocytic lymphadenitis and favor KFD.

There is no evidence to support the diagnosis of lymphoma in this case. Features of Kikuchi disease that may help prevent its misdiagnosis as malignant lymphoma include the following: incomplete architectural effacement with patent sinuses, presence of numerous reactive histiocytic, relatively low mitotic rates, and the absence of Reed-Sternberg cell.

KFD is a self-limited disease, with a rare recurrence (between 3% and 7%). However, according to a study involving Korean patients, adult patients have a high recurrence rate (20.6%), but most symptoms resolve within one to four months [8,14]. There is no definitive cure. However, people with severe symptoms have been treated with glucocorticoids and/or immunoglobulins to relieve the symptoms. Recurrence has been successfully managed with co-administration of glucocorticoids and hydroxychloroquine or with hydroxychloroquine monotherapy as applied in our patients with good results [15]. But observation of the patient with only empirical treatment while waiting for the histological report can be a challenging situation when a malignant condition such as lymphoma is suspected. Thus early investigative measures are crucial so that the right treatment course is followed and anxiety of the patient can be reduced.

## Conclusions

KFD represents a rare cause of lymphadenopathy that must be confirmed with histopathological examination. It is a benign and recurrent condition that mimics malignant lymphoma, and therefore awareness of the disease among physicians is crucial.
